# Diversity of Anaerobic Methane Oxidizers in the Cold Seep Sediments of the Okinawa Trough

**DOI:** 10.3389/fmicb.2022.819187

**Published:** 2022-04-14

**Authors:** Ye Chen, Cuiling Xu, Nengyou Wu, Zhilei Sun, Changling Liu, Yu Zhen, Youzhi Xin, Xilin Zhang, Wei Geng, Hong Cao, Bin Zhai, Jing Li, Shuangshuang Qin, Yucheng Zhou

**Affiliations:** ^1^Key Laboratory of Gas Hydrate, Qingdao Institute of Marine Geology, Ministry of Natural Resources, Qingdao, China; ^2^Laboratory for Marine Mineral Resources, Qingdao National Laboratory for Marine Science and Technology, Qingdao, China; ^3^College of Environmental Science and Engineering, Ocean University of China, Qingdao, China; ^4^Key Laboratory of Marine Chemistry Theory and Technology, Ministry of Education, College of Chemistry and Chemical Engineering, Ocean University of China, Qingdao, China

**Keywords:** ANME, diversity, cold seep, sediment, Okinawa Trough

## Abstract

Active cold seeps in the Okinawa Trough (OT) have been widely identified, but the sediment microbial communities associated with these sites are still poorly understood. Here, we investigated the distribution and biomass of the microbial communities, particularly those associated with the anaerobic oxidation of methane (AOM), in sediments from an active cold seep in the mid-Okinawa Trough. Methane-oxidizing archaea, including ANME-1a, ANME-1b, ANME-2a/b, ANME-2c, and ANME-3, were detected in the OT cold seep sediments. Vertical stratification of anaerobic methanotrophic archaea (ANME) communities was observed in the following order: ANME-3, ANME-1a, and ANME-1b. In addition, the abundance of methyl coenzyme M reductase A (*mcrA*) genes corresponded to high levels of dissolved iron, suggesting that methane-metabolizing archaea might participate in iron reduction coupled to methane oxidation (Fe-AOM) in the OT cold seep. Furthermore, the relative abundance of ANME-1a was strongly related to the concentration of dissolved iron, indicating that ANME-1a is a key microbial player for Fe-AOM in the OT cold seep sediments. Co-occurrence analysis revealed that methane-metabolizing microbial communities were mainly associated with heterotrophic microorganisms, such as JS1, Bathy-1, and Bathy-15.

## Introduction

Methane is an important greenhouse gas known to be 28 times more potent per molecule than carbon dioxide (CO_2_; IPCC, 2014). Continental margins account for only 11% of the ocean area (Levin and Sibuet, 2012); however, their subsurface seabed contains large reservoirs of methane as crystalline gas hydrates, which dissolve and form gaseous methane. Driven by a variety of unstable geological factors, low-temperature, and methane-rich fluids are emitted to the seabed surface along the seabed channels, forming a unique deep-sea ecosystem-cold seep (Boetius and Wenzhoefer, 2013). Cold seeps along the global continental margins emit 0.01–0.05 Gt of carbon to the atmosphere annually, accounting for 1–5% of the global methane emissions to the atmosphere (Niu et al., 2017). A substantial portion of CH_4_ is produced after the degradation of organic matter (DOM) by a consortium of microbes (Ferry and Lessner, 2008). As the major biological sink of methane in marine sediments, the microbially mediated anaerobic oxidation of methane (AOM) plays a crucial role in regulating methane emissions from marine sediments into the hydrosphere. This process is generally linked to sulfate reduction (S-AOM), which establishes a sulfate–methane transition zone (SMTZ) in which methane diffuses from the subsurface and sulfate diffuses from seawater (Iversen and Jorgensen, 1985; Niewöhner et al., 1998; Knittel et al., 2005).

Sulfate reduction-anaerobic oxidation of methane is often mediated by syntrophic interactions between anaerobic methanotrophic archaea (ANME) and sulfate-reducing bacteria (SRB; Boetius et al., 2000; Michaelis et al., 2002; Knittel and Boetius, 2009). ANMEs that are responsible for S-AOM can be divided into three groups: ANME-1 (ANME-1a and ANME-1b), ANME-2 (ANME-2a, ANME-2b), and ANME-3, with internal sequence similarity between the three groups of 75–92% (Knittel and Boetius, 2009). In ANME-1 and ANME-2 archaea, the SRB partner is usually associated with *Desulfosarcina*/*Desulfococcus* (Knittel et al., 2005; Schreiber et al., 2010), while ANME-3 is affiliated with the SRB of the *Desulfobulbus* branch (Niemann et al., 2006; Loesekann et al., 2007). Moreover, ANME-1 is frequently found as single cells, indicating that ANME-1 may perform AOM with sulfate without a bacterial partner (Knittel et al., 2005). In addition to S-AOM, methane can also be oxidized anaerobically using nitrite (Raghoebarsing et al., 2006) or nitrate (Haroon et al., 2013) as alternative electron acceptors. A specific lineage of the ANME-2 clade (ANME-2d) was responsible for nitrate-dependent anaerobic oxidation of methane through reverse methanogenesis using nitrate (Haroon et al., 2013).

Recently, Fe-AOM has been suggested to occur ubiquitously in the methanic zone of iron oxide-rich marine sediments (Riedinger et al., 2014; Egger et al., 2015; Oni et al., 2015; Rooze et al., 2016). Geochemical evidence below the SMTZ, such as high dissolved iron, low to undetectable sulfate, high methane concentrations, and the presence of methane together with large quantities of buried reactive iron oxides, are taken as prerequisites for Fe-AOM (Aromokeye et al., 2020). By measuring rates using incubation experiments, direct evidence of Fe-AOM in marine sediment has been detected in coastal sediments from the Eel River Basin (Beal et al., 2009), the Bothnian Sea (Egger et al., 2015), mud areas from the North Sea (Aromokeye et al., 2020), and methane seeps from the South China Sea (Li et al., 2020a). ANME-1, ANME-3, ANME-2a, ANME-2c, ANME-2d, *Candidatus Methanoperedens ferrireducens Methanosarcina acetivorans*, *Methanobacterium*, or some methanotrophic bacteria were suspected to be related to metal-AOM in various earlier studies (Beal et al., 2009; Ettwig et al., 2016; Scheller et al., 2016; Bar-Or et al., 2017; Cai et al., 2018; Yan et al., 2018; He et al., 2019; Liang et al., 2019). However, the microorganisms responsible for metal-dependent AOM in the marine environment need to be further explored.

The geographic distributions and ecological niches of different ANME subtypes have been found in previous studies. ANME-1 and ANME-2 are widespread in different environments, but ANME-3 is mainly present in mud volcanoes (Felden et al., 2010; Pop Ristova et al., 2012). For the depth profiles of the different ANMEs, ANME-1, and ANME-3 were found to predominate in the deeper sediments, which were anoxic, methane-rich, and sulfate-containing sediments (Knittel et al., 2005). ANME-2c was found to be predominant in the deeper sediments close to gas hydrates, in which methane and sulfide concentrations were higher (Roalkvam et al., 2011). In contrast, ANME-2a/b appeared in the upper sediments with higher sulfate concentrations and lower concentrations of dissolved sulfide and methane (Roalkvam et al., 2011; Yanagawa et al., 2011). Electron acceptor (e.g., SO_4_^2−^ and Fe^3+^) availability and dissolved inorganic carbon have been suggested as crucial factors for shaping the ANME community composition in methane-rich sediments (Niu et al., 2017; Schnakenberg et al., 2021). However, despite recent progress, the extent to which geochemical heterogeneity affects the ecological niches of different ANME clades needs further investigation.

The Okinawa Trough (OT), which is located in the eastern part of the East China Sea (ECS), is an incipient back-arc basin with a total area of ~1.4 × 10^5^ km^2^ (Glasby and Notsu, 2003; Yan and Shi, 2014). Cold seep sites have been widely identified in the OT (Li et al., 2020b). Previous studies on seeps in the OT have led to many significant advancements, including new insight into AOM (Sun et al., 2015, 2019; Peng et al., 2017), mineral isotopes (Cao et al., 2020), pore water composition (Xu et al., 2020), and the molecular biology of cold seep carbonates (Li et al., 2018). For example, shallow SMTZs were detected by pore water analysis in OT cold seeps [within 10 and 40 cm below the seafloor (cmbsf)], indicating that intensive methane seepage and active anaerobic methane oxidation occurred in these areas (Xu et al., 2020). In addition, petrologic and mineralogical observations provided evidence for the presence of metal-AOM (Sun et al., 2015, 2019; Peng et al., 2017). Nevertheless, to our knowledge, the structure of sedimentary microbial communities, particularly those involved in anaerobic oxidation of methane, are uncharacterized in this habitat.

In this study, we investigated the microbial community, especially the anaerobic methane oxidizers (ANME) communities in sediments of the OT cold seep area, with the aim of (i) determining the diversity and vertical distribution of the ANME clades in the cold seep sediments of the OT; (ii) further evaluating the environmental factors controlling the niche separation and distribution of ANME subgroups; and (iii) investigating interactions between ANME archaea and other microbial taxa in the communities present. This study can provide a basic description of the structure and potential function of microbial communities involved in AOM, with the aim of providing a better understanding of the methane cycle in the submarine cold seeps of the OT.

## Materials and Methods

### Sample Collection

**Figure 1:**
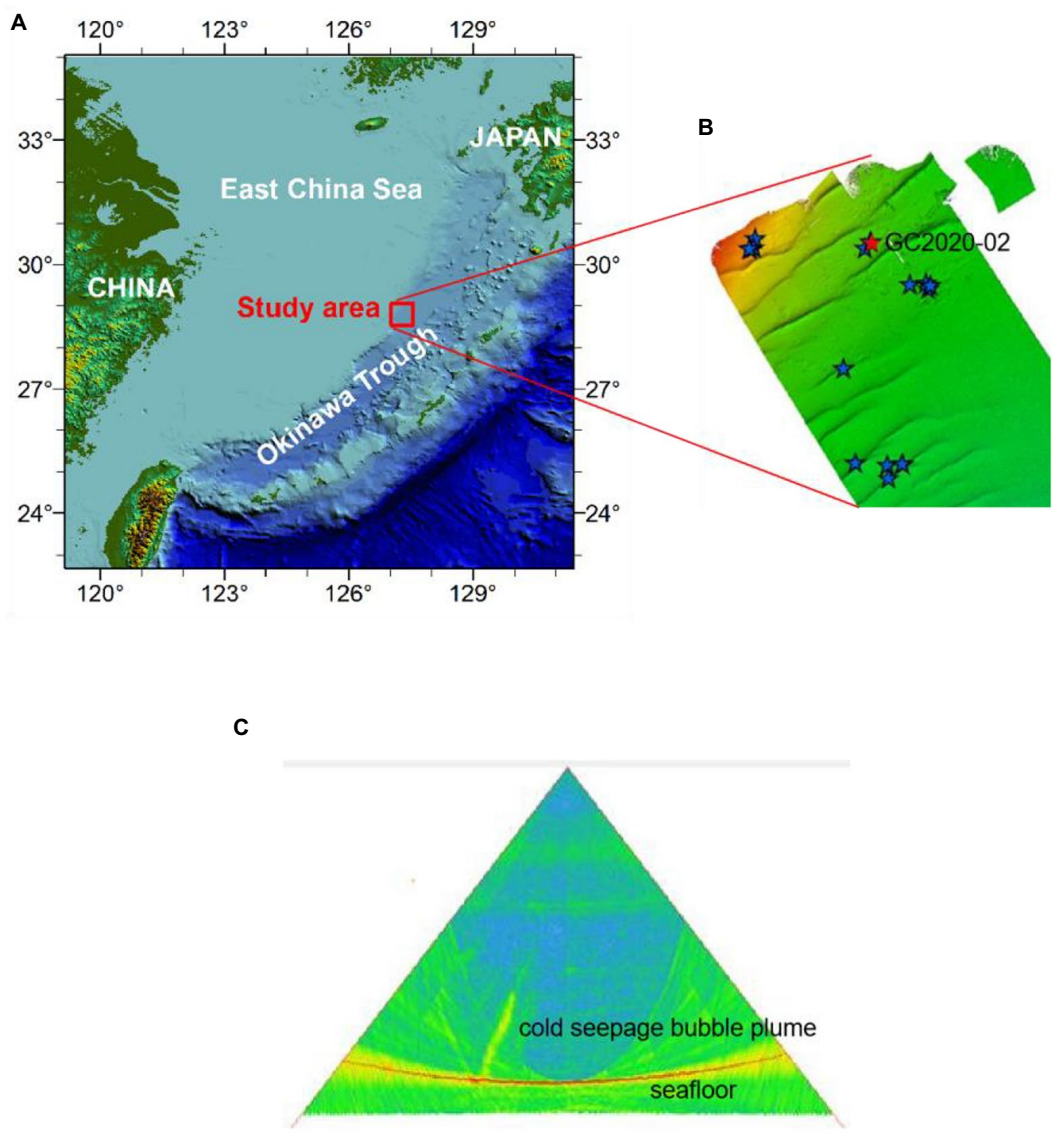
Topographical profile of the Okinawa Trough (OT; **A**), multibeam topographic map **(B)**, and multibeam backscatter image **(C)** of the study area [in the multibeam topographic map **(B)**, the five-pointed stars represent the distribution of acoustic plumes in water, and the red five-pointed stars represent sampling site GC20202-02].

Our study provides a molecular analysis of a 1.45-m-long gravity core taken from the western slope of the mid-Okinawa Trough during the R/V Haiyang Dizhi 9 expedition (August–September 2020; Figure 1A). Core GC2020-02 was located on a seafloor dome structure, where intense methane seepage has been inferred by pore water geochemical data with an SMTZ as shallow as 10 cmbsf (Xu et al., 2020). The acoustic acquisition of the water column using multibeam systems indicated that there were gas bubble emissions at GC2020-02 (Figures 1B,C). To collect the headspace CH_4_, wet sediment was collected using cutoff syringes at a depth interval of 7.5 cm and immediately transferred into 20-ml glass vials filled with 10 ml of saturated NaCl solution, sealed with a rubber stopper, and subsequently stored upside down in a 4°C refrigerator. At the corresponding layer, subsamples for molecular analysis were collected with a sterile tongue depressor and stored at −80°C for nucleic acid extraction. The remaining untouched sediments were pressed onboard at 7.5 cm intervals for geochemical analyses.

### Geochemical Analyses

Sedimentary methane was determined using an Agilent 6,890 N modified gas chromatograph (GC) with a flame ionization detector. The pore water subsample sulfate content was measured by an IC-1600 ion chromatography system (Dionex Corporation, United States). Dissolved trace elements (Fe, Mn) in the pore water samples were assessed using an iCAPQ (Thermo, United States). The concentrations of sulfide were determined with a TU-1901 double light beam ultraviolet obvious spectrophotometer.

### DNA Extraction

Microbial DNA was extracted from 0.3 ~ 0.5 g of sediment using the PowerSoil DNA Kit (MoBio Laboratories, Inc., Carlsbad, CA, United States) according to the manufacturer’s instructions.

### Illumina Sequencing of Microbial 16S rRNA

The bacterial hypervariable V3–V4 and archaeal hypervariable V4–V5 regions of the 16S rRNA gene were amplified using barcoded 338F (ACTCCTACGGGAGGCAGCA)/806R (GGACTACHVGGGTWTCTAAT; Lee et al., 2012) and Arch519 (CAGCCGCCGCGGTAA)/Arch806 (GTGCTCCCCCGCCAATTCCT; Coolen et al., 2004) primers. PCR was carried out in a 25 μl reaction volume with ABI 2720 (Applied Biosystems, United States) with the following cycling parameters: initial predenaturation at 98°C for 5 min, followed by 25 cycles of denaturation at 98°C for 30 s, annealing at 55°C (V3–V4) or 65°C (V4–V5) for 30 s, elongation at 72°C for 45 s, and a final extension at 72°C for 5 min. The PCR mixture contained 0.25 μl of Q5 High-Fidelity DNA Polymerase, 5 μl of Q5 Reaction Buffer (5×), 5 μl of Q5 High-Fidelity GC Buffer (5×), 2 μl of dNTP Mix (2.5 mM), 1 μl of each primer (10 μM), 2.0 μl of template, and 8.75 μl of ddH2O. Amplicons were purified using the PicoGreen dsDNA Assay Kit (Invitrogen, United States) according to the manufacturer’s instructions and sequenced using the Illumina MiSeq PE250 platform (Illumina, United States).

### Clone Library Construction for *mcrA* Genes

Methyl coenzyme M reductase A genes (*mcrA*) associated with methanogens and ANME were amplified with primers ME1 (GCMATGCARATHGGWATGTC) and ME2 (TCATKGCRTAGTTDGGRTAGT; Hallam et al., 2003). PCR was performed using the following procedure: denaturation at 98°C for 5 min followed by 40 cycles of denaturation at 98°C for 15 s, annealing at 50°C for 30 s, and elongation at 72°C for 1 min, followed by a final elongation step at 72°C for 10 min. Amplicons encoding the *mcrA* gene were purified with the Ultra Clean GelSpin DNA Purification kit (Takara, Dalian, China) according to the manufacturer’s instructions. PCR products were ligated to the pMD18-T vector (TaKaRa) and then transformed into competent *Escherichia coli* DH5α cells. Transformants were plated onto LB/Amp/X-Gal/IPTG plates and incubated overnight at 37°C. Positive colonies were selected and sequenced by the Beijing Genomics Institute (BGI, Beijing, China).

### Quantification of the *mcrA*, *dsrB*, and 16S rRNA Genes

Methyl coenzyme M reductase A genes were quantified using the general primers mlasF/ME2mod (Steinberg and Regan, 2008; Miyazaki et al., 2009). Moreover, the group-specific primers mcrA_ab_fw/mcrA-ab-rv and mcrA_ab_fw/mcrA-ab-rv were used to target the *mcrA* genes of ANME-1 and ANME-3 (Miyazaki et al., 2009), respectively. The total bacterial 16S rRNA gene, archaeal 16S rRNA gene, and *dsrB* gene were amplified using the primers 338F/806R, Arch519/Arch806, and DSRp2060F/DSR4R (Geets et al., 2006), respectively. All PCR assays were performed with an ABI PRISM® 7500 Sequence Detection System (Applied Biosystems, United States). Each 20-μl mixture contained 10 μl FastStart Universal SYBR Green Master Mix (Rox) (Roche Diagnostics, Germany), 2.0 μl sediment DNA, 0.6 μl each forward and reverse primer (10 μM), 0.2 μl bovine serum albumin (BSA), and 6.6 μl ddH_2_O. Standard curves were generated using 10-fold dilutions of plasmids carrying the target gene fragments. Each reaction was conducted in triplicate. The specificity of PCR amplification was confirmed by melting curve analysis. The qPCR primers, annealing temperatures, and assay conditions are listed in Supplementary Table S1.

### Analysis of 16S rRNA and *mcrA* Gene Sequences

To analyze the 16S rRNA gene sequences, raw reads were trimmed and cleaned by removing adaptor sequences using the Divisive Amplicon Denoising Algorithm 2 (DADA2) within the QIIME2 package (version 2019.4; Callahan et al., 2016). After data filtering was performed, unique amplicon sequence variants (ASVs) were derived and classified *via* the Dada2 pipeline. Taxonomy assignment for ASV was performed using the SILVA 16S rRNA database (version 138). To fairly compare microbial community compositions and diversity at equal sequencing depths, 95% of the minimum sequence numbers (37,654 for archaea and 26,512 for bacteria) among all samples were randomly selected from each sample. Finally, read numbers in each sample were limited to 35,771 and 25,186 sequences for the archaeal and bacterial communities, respectively. Good’s coverage and alpha diversity indices (Chao 1 and Shannon) were also calculated in the QIIME 2 package.

The clone sequences were grouped into operational taxonomic units (OTUs) based on 97% similarity using the Mothur software package. Phylogenetic trees of the representative OTU sequences for the *mcrA* gene were built by maximum likelihood analysis with MEGA software. Confidence in the topology of this tree was evaluated using 1,000 bootstrap replications.

### Statistical Analyses

Microbial co-occurrence network analysis was conducted using the R package psych (V.4.1.0). Bacterial and archaeal ASVs with a relative abundance of more than 1% in at least one sediment sample were first selected to generate co-occurrence patterns. The absolute abundance was determined as their relative abundance in DNA sequence libraries multiplied by archaeal and bacterial DNA quantification. A Pearson coefficient greater than 0.75 and a significance level less than 0.005 indicated a significant correlation. Finally, a network diagram was built using Gephi software (version 0.9.2; Bastian et al., 2009). Redundancy analysis (RDA) with the vegan R package (R version 4.1.0) was used to analyze the relationships between environmental parameters and the distribution of archaeal communities. This analysis was calculated based on the Hellinger transformation of the relative abundance of microbial groups and the original environmental variable data (Borcard et al., 2018). The explained variation *R*^2^ obtained was adjusted using the RsquareAdj function in the vegan package. The significance of the environmental variables was tested by ANOVA based on 999 permutations. A heatmap of the Pearson correlation of ANME and SRB was generated in R software.

## Results

### Core Description and Sediment Geochemistry

**Figure 2:**
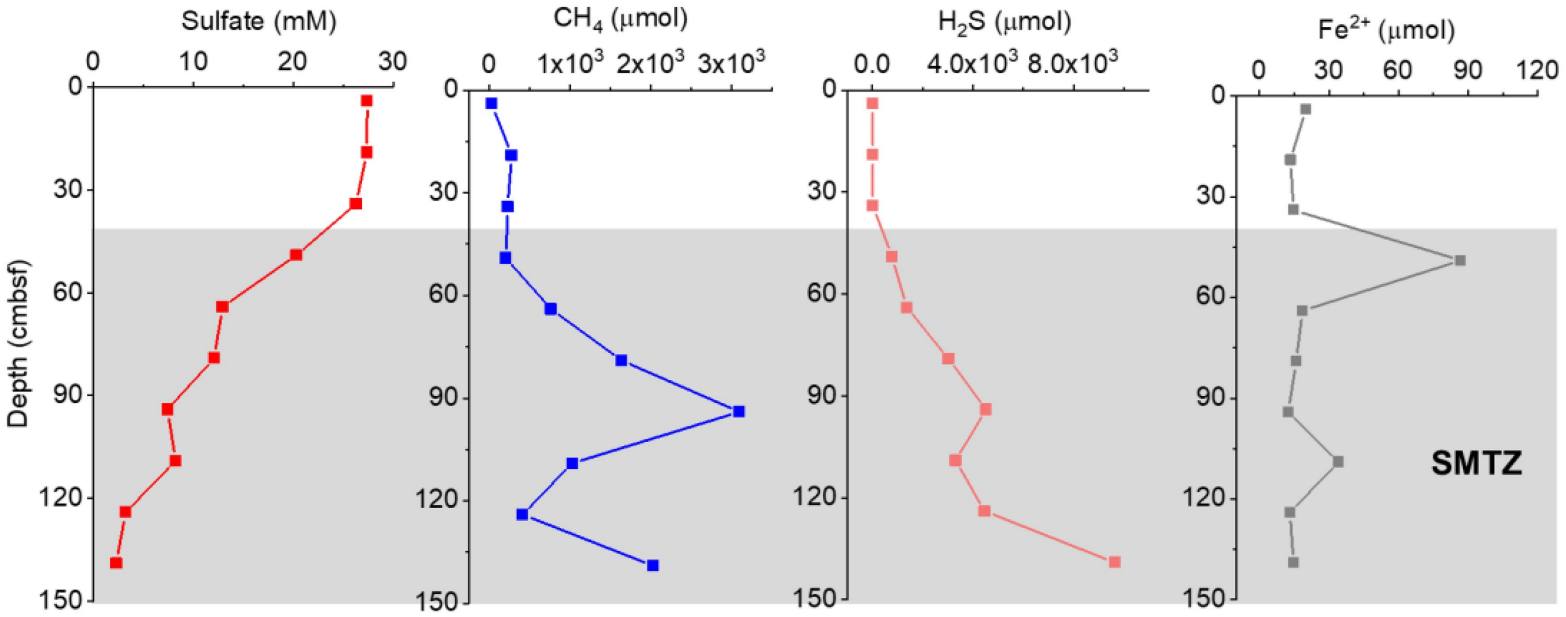
Geochemical profiles for the concentrations of sulfate, CH_4_, H_2_S, and Fe^2+^ in the GC2020-02 core.

At a water depth of 956 m, a 1.45-m-long piston core was retrieved from the OT. A strong sulfide odor was also detected during core processing, which is indicative of a highly reducing environment. The dominant lithology of the sediment from this core was a gray–black silt clay, and carbonate gravels and giant clam shells could be observed in multiple layers. The sulfate, methane, sulfide, and Fe^2+^ contents in the pore water samples are shown in Figure 2. Sulfate concentrations in the pore water decreased with depth, from 27.43 mM at the top of the core to 2.30 mM at the bottom sediment (139 cmbsf). In contrast, the sulfide concentrations showed a significant increase in depth from 19.63 μM at 34 cmbsf to the highest concentration of 9,646 μM at 139 cm depth. A decrease in sulfate showed a strong correlation (*R* = −0.988, *p* < 0.01) with an increase in hydrogen sulfide, indicating that hydrogen sulfide was produced during sulfate reduction. The methane concentrations increased significantly with depth from 761.24 μM at 64 cmbsf to 3091.59 μM at 94 cmbsf and then decreased with depth. In addition, pore water concentrations of dissolved reduced iron (Fe^2+^) ranged from 12.40 to 86.67 μM, with a maximum value detected at 49 cm depth. We have always considered the abnormal maximum value of Fe^2+^ to be reliable since the collection, storage, and analysis of Fe^2+^ for all our pore water samples were conducted in the same way. This iron anomaly could indeed occur because the modern hydrothermal activities in the spreading center of the OT would provide abundant reactive metals to the sediments of the cold seep sites (Sun et al., 2019). Based on the sulfate, methane, and sulfide profiles, the SMTZ of GC2020-02 was likely located below 49 cmbsf (Figure 2). The detailed geochemical profiles of GC2020-02 will be published elsewhere (Xu et al., in preparation).

### Quantification of Gene Abundance

**Figure 3:**
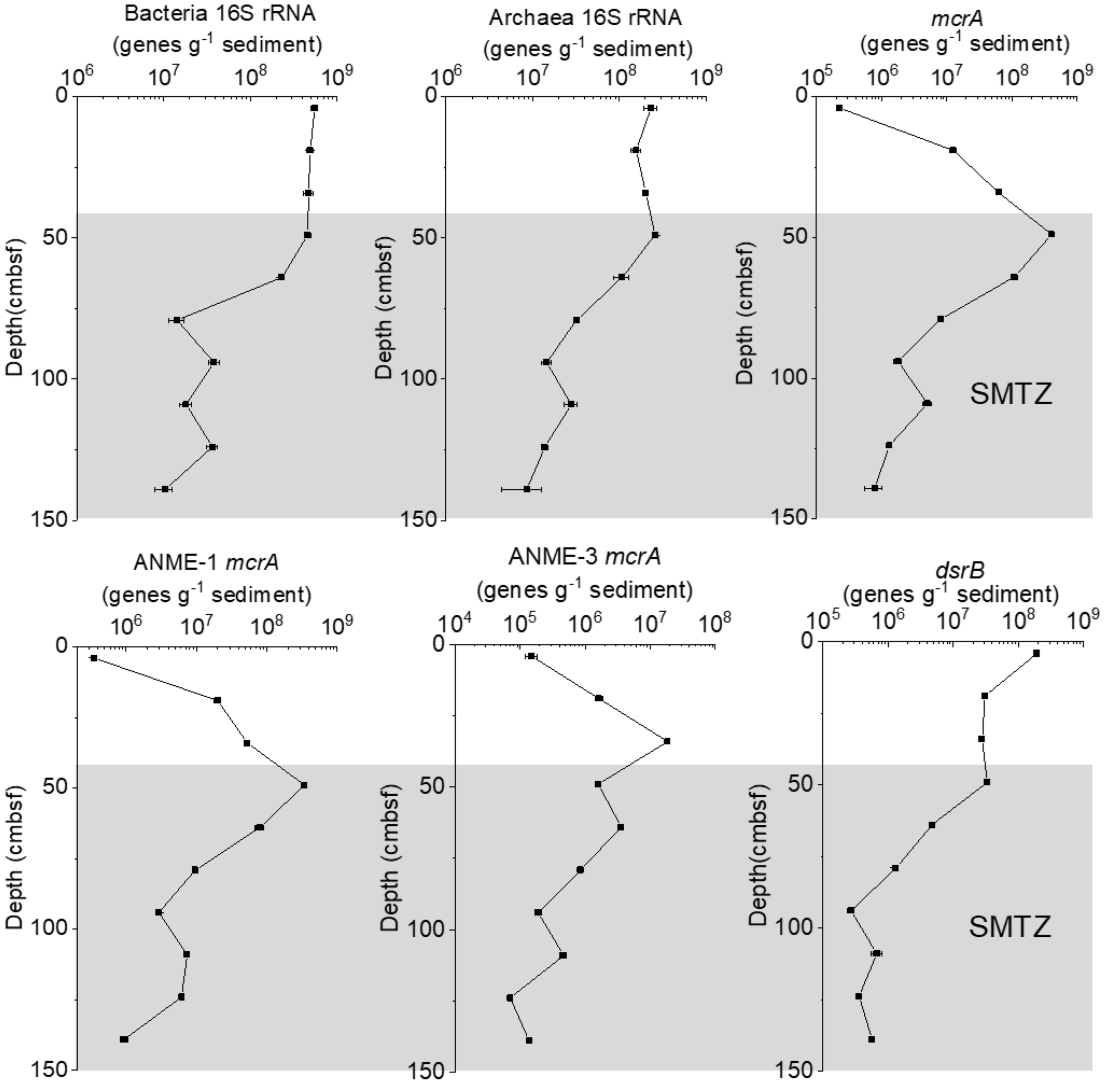
Gene abundance of the bacterial 16S rRNA, archaeal 16S rRNA, *mcrA*, ANME-1 *mcrA*, ANME-3 *mcrA*, and *dsrA* genes in the GC2020-02 core.

The abundance of bacterial 16S rRNA genes was found to range from 1.0 × 10^7^ to 5.5 × 10^8^ genes per gram of wet sediment, while for archaea, it was slightly lower, from 8.5 × 10^7^ to 2.6 × 10^8^ genes g^−1^ (Figure 3). The relative abundance of bacteria in the total microbial community was 30.90–75.92%, and the relative abundance of archaea within the total microbial community was 24.08–69.10%. In the GC2020-02 core, *mcrA* gene abundance ranged from 2.3 × 10^5^ to 4.1 × 10^8^ genes g^−1^ wet weight sediment, while the abundance of the *dsrB* gene ranged from 2.7 × 10^5^ to 1.9 × 10^8^ genes g^−1^ wet weight sediment. The *mcrA* gene abundance of ANME-1 and ANME-3 ranged from 3.5 × 10^5^ to 3.5 × 10^8^ genes g^−1^ wet weight sediment and from 7.1 × 10^4^ to 1.9 × 10^7^ genes g^−1^ wet weight sediment, respectively. The abundances of bacterial, archaeal 16S rRNA, and *dsrB* genes decreased with depth, especially in the estimated SMTZ. In contrast, *mcrA* and ANME-1 *mcrA* gene abundance increased with depth in the upper 49 cmbsf and then decreased sharply with depth. ANME-3 *mcrA* gene abundance increased with depth in the upper 34 cmbsf and then decreased rapidly with depth.

### Archaeal and Bacterial Diversity Analysis

A total of 488,786 and 749,445 high-quality sequences from archaea and bacteria were recovered for the 10 seep sediment samples, which were clustered into a total of 15,049 archaeal ASVs and 21,888 bacterial ASVs, respectively (Supplementary Table S2). Good’s coverage of archaeal and bacterial communities ranged from 97.68–99.64% to 94.27–99.19%, respectively, which indicated that a majority of the archaeal and bacterial taxa in the sediments had been covered (Supplementary Table S2). Generally, lower archaeal and bacterial alpha diversity indices, including Chao 1 and Shannon, were detected in the SMTZ sediment than in the sediment layers above the SMTZ (Supplementary Table S2). In the GC2020-02 core, Bathyarchaeota (30.20–50.01%), Methanomicrobia (0.11–46.51%), Lokiarchaeia (1.42–15.67%), and Thermoplasmata (4.27–26.54%) were the major archaeal members (Supplementary Figure S1A). Bathyarchaeota was the dominant archaeal member across most sediment depths; additionally, the methane-metabolizing archaea Methanomicrobia mainly dominated the estimated SMTZ and had the highest relative abundances in the 49 and 64 cm layers (Supplementary Figure S1A). For the bacterial community, the sediment layers (4–34 cm) above the estimated SMTZ contained Deltaproteobacteria, Dehalococcoidia, and Anaerolineae (Supplementary Figure S1B). In the estimated SMTZ sediment (49–139 cm), JS1 was the most dominant bacterial group, accounting for 23.13–59.87% of the total bacterial sequences obtained (Supplementary Figure S1B).

### Composition of Methane-Metabolizing Archaea and Sulfate-Reducing Bacteria

**Figure 4:**
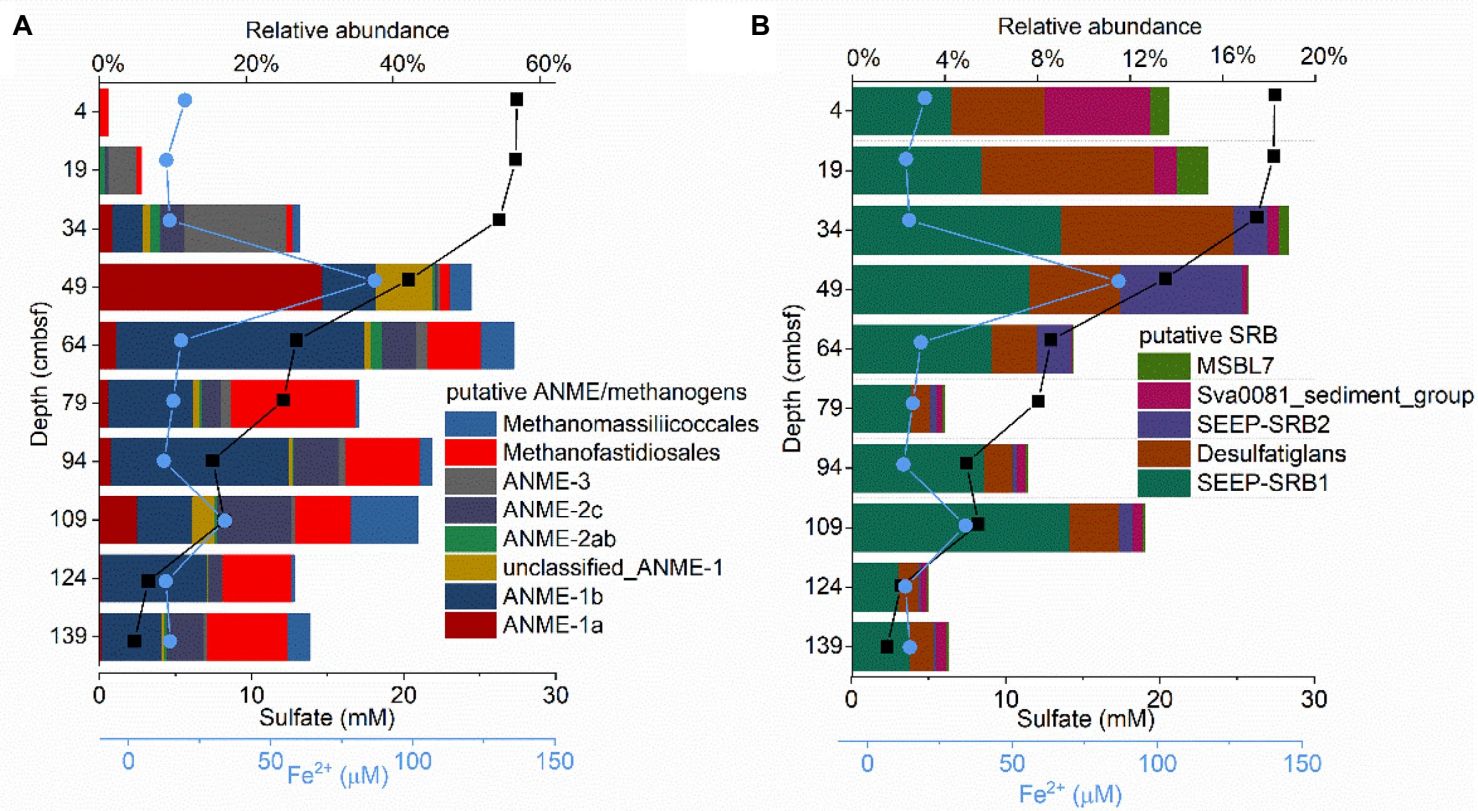
The composition of putative anaerobic methanotrophic (ANME)/methanogens **(A)** and putative sulfate reducing bacteria (SRB; **B**) obtained from the 16S rRNA gene sequences from the GC2020-02 core.

Sequences affiliated with putative methanogens and putative ANME, which represent 8.89 and 23.14%, respectively, of the total archaeal sequences, were abundant in GC2020-02. Methanogens and ANME were present in considerably lower proportions in the surficial sample (4 cm), became highly abundant at 49 and 64 cm, and decreased with sediment depth (Figure 4A). Taxonomic classification revealed that the GC2020-02 sediment methanogens mainly belonged to Methanofastidiosales and Methanomassiliicoccales (Figure 4A). Methane-oxidizing archaea within the GC2020-02 sediments included ANME-1a, ANME-1b, ANME-2ab, ANME-2c, and ANME-3 (Figure 4A). The methane-metabolizing microbial community composition varied among sediment depths. Methanogens associated with Methanofastidiosales and Methanomassiliicoccales were mainly dominant in the sediment layers of 49–139 cmbsf. For the anaerobic methane-oxidizing archaeal groups, ANME-3 dominated in the upper layers (19 and 34 cm) with high concentrations of sulfate and was decreased and replaced by ANME-1a in the 49 cm layer. Then, the relative abundance of ANME-1a decreased, but ANME-1b was enriched in the deeper sediments (64–139 cmbsf). The sequences affiliated with ANME-2c were mainly detected below 34 cm, in which there was a decrease in sulfate with a concomitant increase in sulfide and methane. Sequences obtained from the V3–V4 region were also used to analyze the diversity and composition of the methanogen/ANME community. The ANME communities were dominated by ANME-1a, followed by ANME-1b, ANME-2ab, ANME-2c, and ANME-3. The changing trend of the relative abundances of ANME subgroups along the vertical profile was consistent with the results from the analysis of V4-V5 (Supplementary Figure S2). Minor sequences affiliated with the methanogen Methanosaeta were detected at a sediment depth of 64 cm. In addition, *mcrA* gene clone libraries for the seven sediment layers were constructed and analyzed. In total, 291 *mcrA* gene sequences were obtained from the GC2020-02 core and classified into *mcrA* groups a, b, c, d, e, and f (Supplementary Figure S3).

Taxonomic classification of bacterial reads revealed that SEEP-SRB1, with relative abundances ranging from 2 to 9% of the total bacterial sequences, was the most dominant SRB community (Figure 4B). Desulfatiglans was the second most dominant SRB community, and this group was hosted mainly in the upper layers of GC2020-02 (Figure 4B). Additionally, a high relative abundance of Sva0081_sediment_group (5%) was detected at 4 cmbsf, and a high relative abundance of SEEP-SRB2 (5%) was detected at 49 cmbsf (Figure 4B).

### The Relationship Between Methane-Metabolizing Archaea and SRB

**Figure 5:**
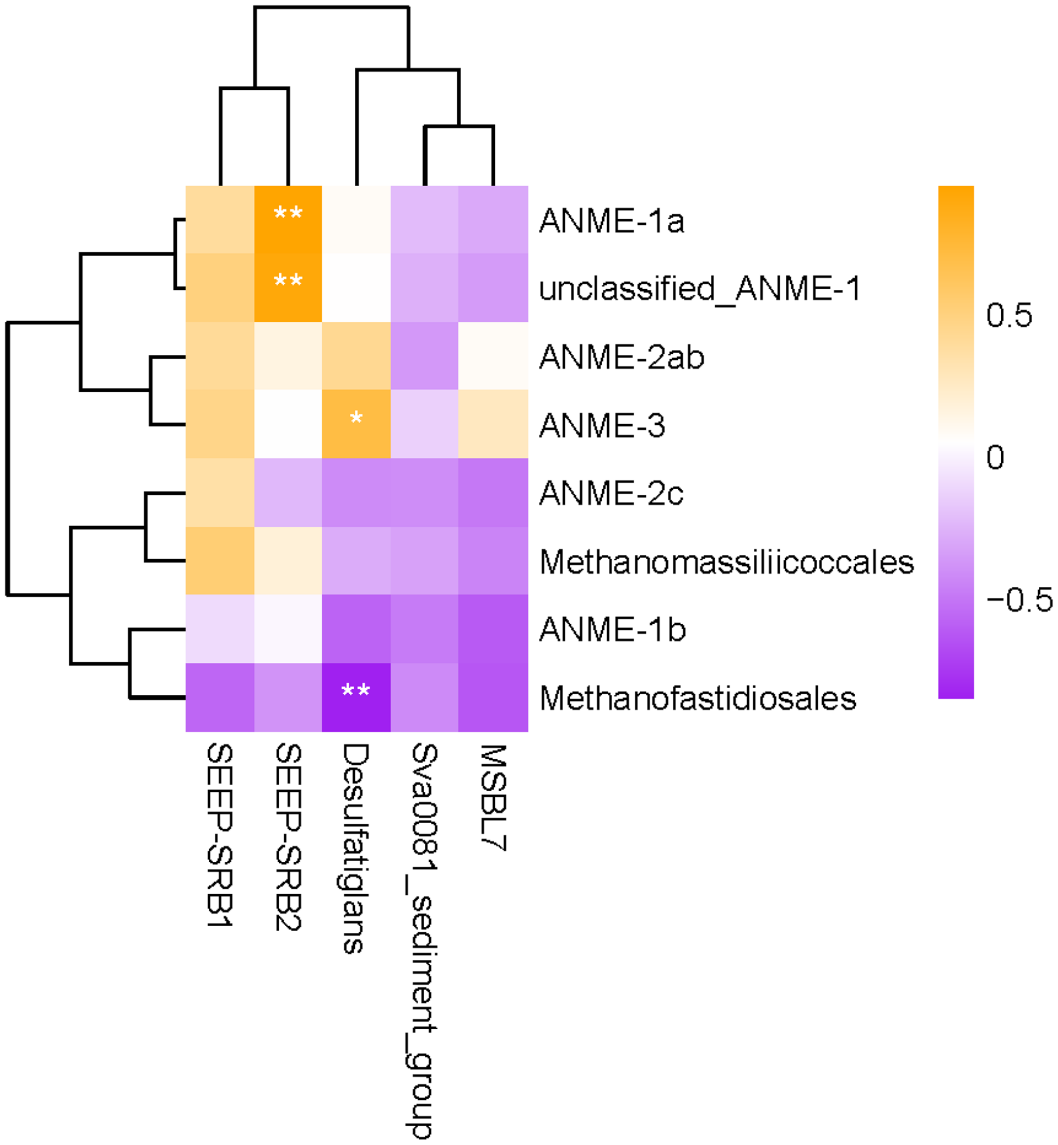
Heatmap of Pearson’s correlation coefficients between methanogens/ANMEs and SRB. Correlation coefficients with *p*-value scores less than 0.05 and 0.01 are labeled with “*” and “**”, respectively.

To better understand the coupling between anaerobic methane oxidation and sulfate reduction in this location, we performed Pearson’s correlation analysis to explore the possible relationship between methane-metabolizing archaea and SRB. The results showed that the relative abundances of ANME-1a and some other ANME-1 groups were positively correlated with SEEP-SRB2 in sediments (*p* < 0.01; Figure 5). The relative abundance of ANME-3 was positively correlated with that of Desulfatiglans in sediments (*p* < 0.05). In addition, the relative abundance of Methanofastidiosales was significantly negatively correlated with Desulfatiglans (*p* < 0.01).

### Microbial Network Analysis

**Figure 6:**
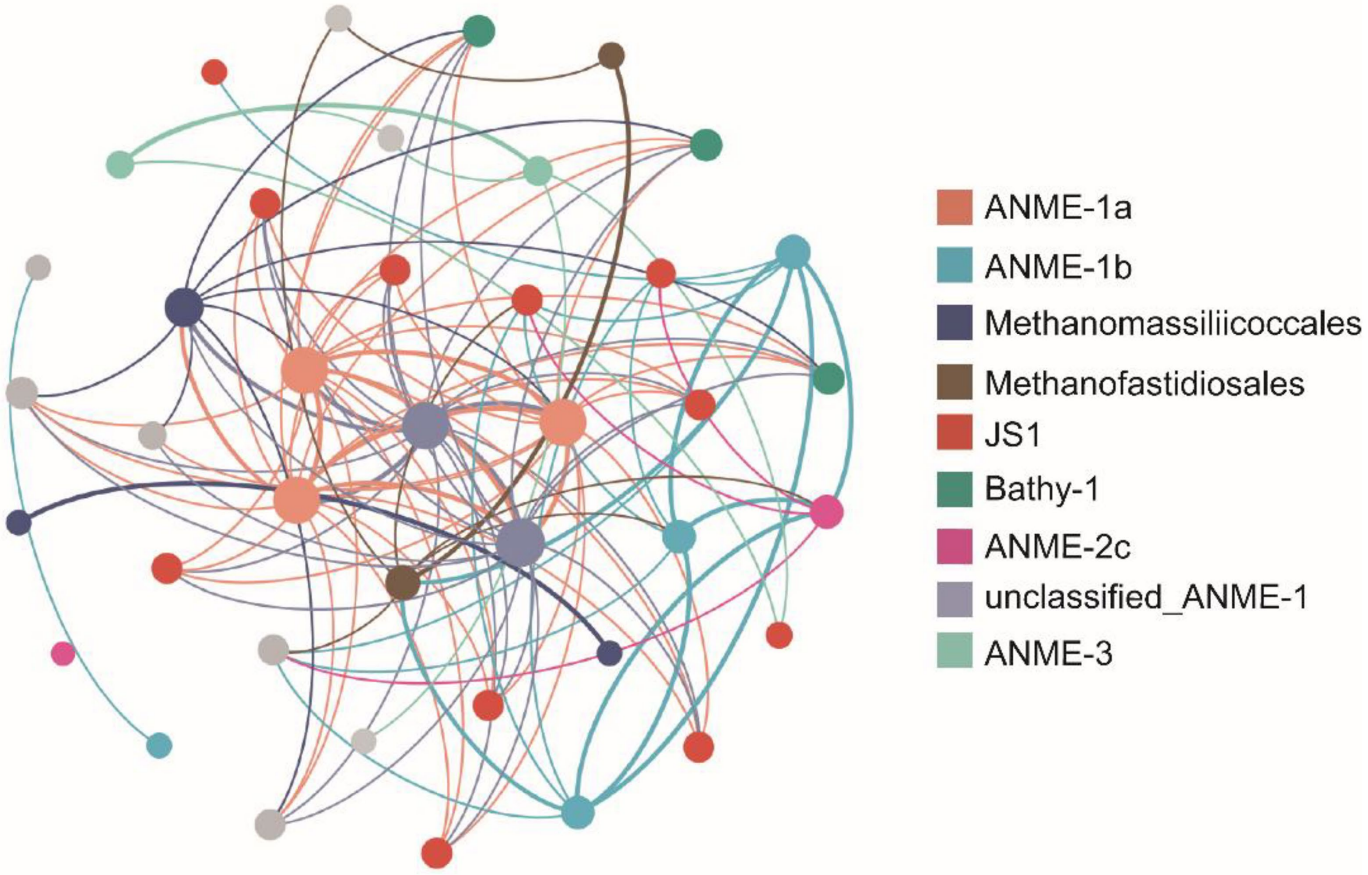
Network co-occurrence patterns of the dominant amplicon sequence variants (ASVs) regarding the relationship of the methane-metabolizing archaea with other microbial taxa in the GC2020-02 core. Only those with a Pearson coefficient > |0.75| and a value of *p* < 0.005 between ASVs are shown in the figure to reduce complexity. A node represents an ASV, and the node size reflects the number of connections the ASVs have with other ASVs. Edges are colored according to the colors of the nodes that they link, and the edge thickness indicates the strength of the correlation.

To provide insight into the biotic interactions, co-occurrence network patterns of seep archaeal and bacterial communities were determined based on Pearson correlation analysis (Figure 6). The subnetworks regarding relationships between the methane-metabolizing archaea and other microbial taxa were selected. Methane-metabolizing archaea all showed significant correlations with other microbial taxa. ANME-1a (three nodes) was most connected with microorganisms within JS1 but was also connected with Bathy-1, Methanofastidiosales, and Methanomassiliicoccales. Four nodes belonging to ANME-1b were most related to JS1 and the methanogen Methanofastidiosales. One ASV belonging to ANME-2c was related to JS1 and Methanofastidiosales. Two nodes belonging to ANME-3 preferred to be connected with microorganisms within JS1 and Bathy-1. ASVs belonging to the methanogen groups (Methanofastidiosales and Methanomassiliicoccales) were mainly connected with ANME clades, such as ANME-1a, ANME-1b, and unclassified_ANME-1. The correlation between ANME clades and methanogen groups implied that a certain degree of coupling between methane production and oxidation was observed in the seep sediments.

### Environmental Factors Shaping the Distribution of the Archaeal Community

**Figure 7:**
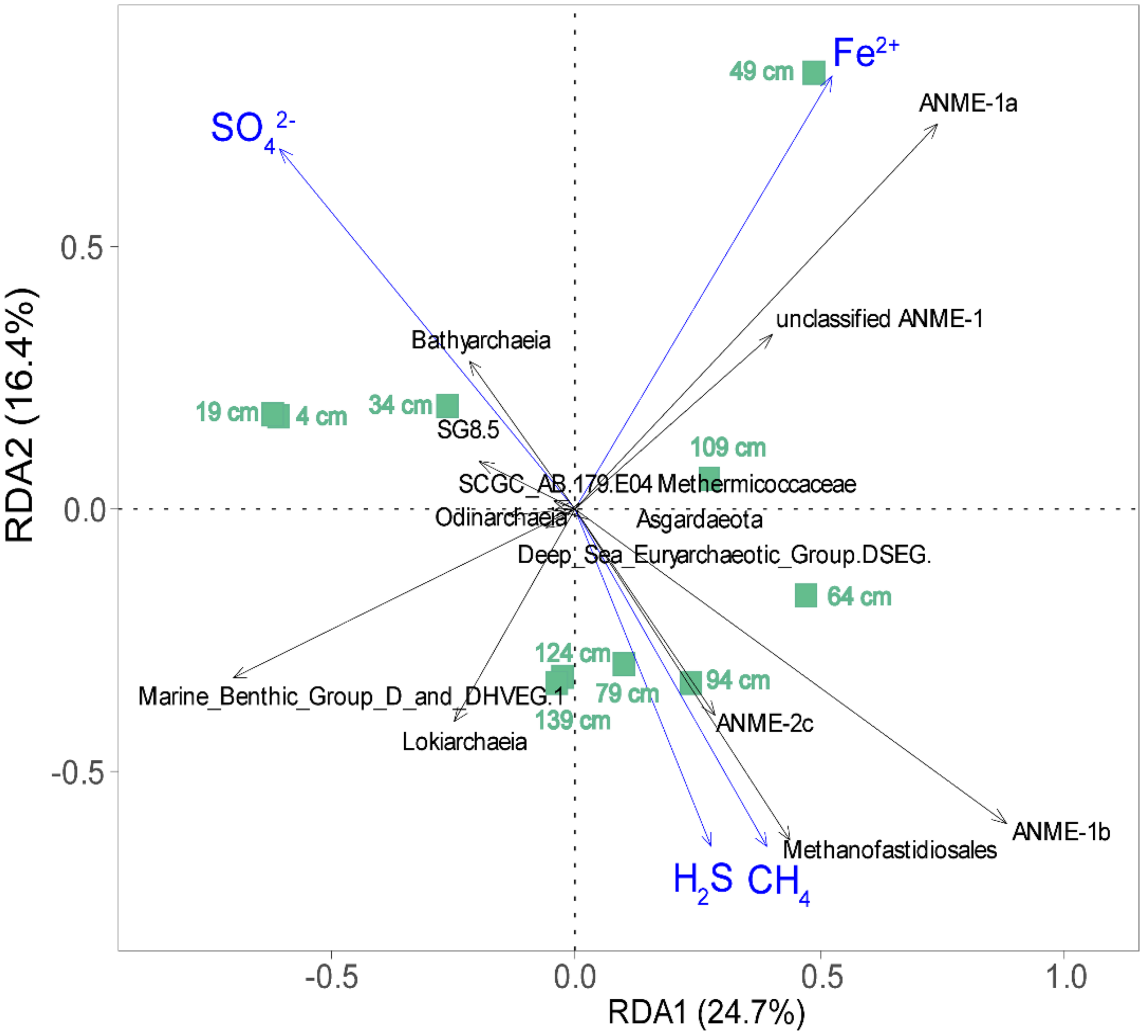
Redundancy analysis (RDA) ordination plot for the first two principal dimensions of the relationships between archaeal community structure and environmental variables (sulfate, CH_4_, H_2_S, and Fe^2+^) in this study. The green square represents 10 detected sediment samples in the OT cold seep sediments; environmental parameters and microbial taxa with a goodness of fit of at least 0.45 are represented as blue arrows and black arrows, respectively.

Redundancy analysis was performed to gain a better understanding of the influence of environmental parameters on the taxonomic composition of ANME and methanogens (Figure 7). For clarity, only those archaeal taxa with high goodness-of-fit values (≥0.45) were included in our RDA plot. The environmental factors (sulfate, CH_4_, H_2_S, and Fe^2+^) accounted for 41.1% (adjusted *R*^2^) of the variation in the archaeal communities, as determined through RDA (Figure 7). Fe^2+^, SO_4_^2−^, and H_2_S accounted for 25.5, 16.0, and 4.8% of the variance in archaeal community composition, respectively (Figure 7). ANOVA showed that Fe^2+^ (*p* < 0.01) and SO_4_^2−^ (*p* < 0.05) significantly contributed to the heterogeneous distribution of major archaeal clades. ANME-1a showed a stronger correlation with Fe^2+^, while ANME-1b and Methanofastidiosales correlated strongly with methane. In addition, ANME-1b and ANME-2c correlated strongly with sulfate and occurred preferentially in the low-sulfate sediment (Figure 7).

## Discussion

Anaerobic oxidation of methane in marine sediment is an important filter that prevents cold seep methane release from marine sediments into the hydrosphere. S-AOM performed by consortia of ANME and SRB in the SMTZ efficiently filters 90% of the diffusing methane (Reeburgh, 2007; Knittel and Boetius, 2009). In addition to S-AOM, iron oxides have been suggested to serve as additional electron acceptors for AOM (Beal et al., 2009), and it has been suggested that Fe(III)-dependent AOM (Fe-AOM) potentially represents a major global methane sink (Cai et al., 2018). However, the microbes involved and the microbial mechanisms for Fe-AOM in the marine environment are still enigmatic. Previous studies indicated that ANME communities can be significantly influenced by geochemical parameters, such as the availability of electron acceptors (Schnakenberg et al., 2021), temperature (Ruff et al., 2015), methane flow intensity (Niemann et al., 2006), dissolved inorganic carbon (Niu et al., 2017), or sulfate (Biddle et al., 2012; Niu et al., 2017). Here, we investigate the diversity of methane-metabolizing archaeal groups and evaluate the environmental parameters that impact the distribution of these archaeal groups in the sediments of an active seep from the OT, which is located in the eastern part of the ECS.

### Diversity of Methane-Metabolizing Microbes in OT Seep Sediments

*Methyl coenzyme M reductase A* genes in the seep sediments of the OT from the surface to bottom layers were between 10^5^ and 10^8^ genes g^−1^ (wet weight), which was higher than the abundances observed in the Haima cold seep (10^3^–10^7^ genes g^−1^; Niu et al., 2017), northern continental slope of South China Sea (10^3^–10^5^ genes g^−1^; Fan et al., 2017), and cold seeps of the Northern South China Sea (10^5^ genes g^−1^; Zhang et al., 2020). In addition, we used 16S rRNA and *mcrA* genes to investigate the diversity of methane metabolic microbes. According to the 16S rRNA gene analysis, methanogens and ANME in the GC2020-02 sediment core included Methanofastidiosales, Methanomassiliicoccales, ANME-1a, ANME-1b, ANME-2a/b, ANME-2c, and ANME-3 (Figure 4A; Supplementary Figure S2). A clone library of the *mcrA* gene demonstrated that the GC2020-02 core contains *mcrA* groups a–b, c–d, e, and f (Supplementary Figure S3). The *mcrA* groups a–b, c–d, e, and f are phylogenetically congruent with ANME-1, ANME-2c, ANME-2a, and ANME-3, respectively (Knittel and Boetius, 2009). In the present study, all *mcrA* gene sequences obtained from GC2020-02 were distributed in ANME groups, which was consistent with the ANME taxonomic profiles produced by 16S rRNA gene amplicon sequencing. However, methanogen groups (Methanofastidiosales and Methanomassiliicoccales) were not detected in GC2020-02 based on *mcrA* gene analysis. A known problem is that some *mcrA*-targeting primer sets are known to exclude certain methanogen taxonomic groups, for example, the *mcrA3* set is unable to detect members of Methanosaetaceae (Nettmann et al., 2008; Ma et al., 2013). *In silico* analysis^1^ revealed that the ME primer does not contain Methanofastidiosales and Methanomassiliicoccales. Thus, we could not detect these two main methanogens in the studied cold seep sediments when the ME primer set was used.

The presence of ANME-1, ANME-2, and ANME-3 is restricted to anoxic, methane-rich, and sulfate-containing sediments (Knittel et al., 2005). Cold seep sediments are highly reduced, and oxygen typically diffuses from only a few millimeters to centimeters into the sediment (Ruff et al., 2015). Thus, cold seep sediment provides an ideal habitat for various subgroups of the ANME. The co-occurrence of three ANME clades (ANME-1, ANME-2, and ANME-3) was detected in the OT cold seep. To identify the geographical distribution of ANME clades, the diversity of ANME communities in cold seep systems distributed on continental margins worldwide is summarized in Supplementary Table S3. ANME-1 and ANME-2 appeared to occur in a wide variety of seep environments, while ANME-3 existed occasionally in marine methane seeps, where methane was vigorously emitted, such as methane hydrate at Hydrate Ridge (Knittel et al., 2005), the Sonora Margin cold seeps (Vigneron et al., 2013), and the Haakon Mosby Mud Volcano (Loesekann et al., 2007). At site GC2020-02, the ANME-1 clade was identified as the most abundant taxon of the anaerobic methane-oxidizing community. A continuous-flow bioreactor incubation experiment showed that ANME-1 archaea flourish at high methane flow rates, whereas ANME-2 archaea are more active at lower flow rates (Girguis et al., 2005). The presence of ANME-3 and higher abundance of ANME-1 at site GC2020-02 strongly indicated that high methane fluxes might occur in this habitat, which was in accordance with field observations and geophysical evidence.

Members in the ANME-1, ANME-2a, b, c, and ANME-3 clades are recognized as sulfate-AOM mediators (Knittel and Boetius, 2009). These archaea often form consortia with SRB to catalyze S-AOM. In the present study, the relative abundance of ANME-1a and some other ANME-1 groups showed a significant correlation with that of SEEP-SRB2 (*p* < 0.01), indicating that the ANME-1 and SEEP-SRB groups may have formed AOM aggregates in the OT cold seep sediments. In addition, ANME-1 could mediate AOM without a bacterial partner in sulfate-poor sediments, as suggested previously (Orphan et al., 2002; Maignien et al., 2013).

### Depth Profile of ANME Clades in the OT Cold Seep

Ecological transitions of ANME-3-, ANME-1a-, and ANME-1b-dominated communities were observed with increasing depth, indicating that ANME subgroups have distinct niche-specific stratification in this habitat (Figure 4A). ANME-3 appeared in surficial, sulfate-rich sediments of site GC2020-02. This observation is consistent with previous results, which indicate that the ANME-3 members were always enriched in methane seep sediments near the surfaces containing comparatively higher sulfate concentrations (Knittel et al., 2005; Miyazaki et al., 2009; Vigneron et al., 2013). Surface colonizers (*Beggiatoa* mats) were considered an influential factor in determining the distribution of ANME-3 (Loesekann et al., 2007; Vigneron et al., 2013). In contrast, ANME-1 was detected in a larger proportion in GC2020-02 sediments and was mainly dominant at the estimated SMTZ. Combined with geochemical data, the results indicated that ANME-1 tended to dominate completely anoxic, sulfate-depleted and highly sulfidic sediments (Knittel and Boetius, 2009; Miyazaki et al., 2009; Yanagawa et al., 2011; Biddle et al., 2012; Niu et al., 2017), which indicated that ANME-1 ecophysiology could depend on environmental conditions (Vigneron et al., 2013). Moreover, we also found that niche separation of ANME-1 subgroups with ANME-1a was predominant in the upper layer (48.75 cm) of the estimated SMTZ, whereas ANME-1b outcompeted ANME-1a in the sulfate-depleted bottom layers (63.75–123.75 cm) of the estimated SMTZ. Consistent with our observations, a previous report showed that in Haima cold seeps of the South China Sea, ANME-1b archaea were dominant in highly sulfate-depleted sediments (Niu et al., 2017). Although the ecological niches of ANME-1a vs. ANME-1b remain unclear, our findings suggest that the distribution of ANME-1a was most influenced by dissolved iron (Fe^2+^), while ANME-1b appeared to prevail in deeper, more sulfate-depleted, higher methane and sulfidic sediments. In addition, ANME-2c dominance occurred in the estimated SMTZ of site GC2020-02 with decreased sulfate and increased sulfide concentrations and methane flux. This result is generally consistent with the observations that in the Nyegga area, ANME-2c thrived in deeper sediment layers where the methane flux and sulfide concentration were relatively high (Roalkvam et al., 2011). The coexistence of ANME clades with very different habitat preferences at site GC2020-02 strongly suggests that they may possess diverse metabolic capabilities.

### Evidence for the Possible Occurrence of Fe-AOM in the OT

**Figure 8:**
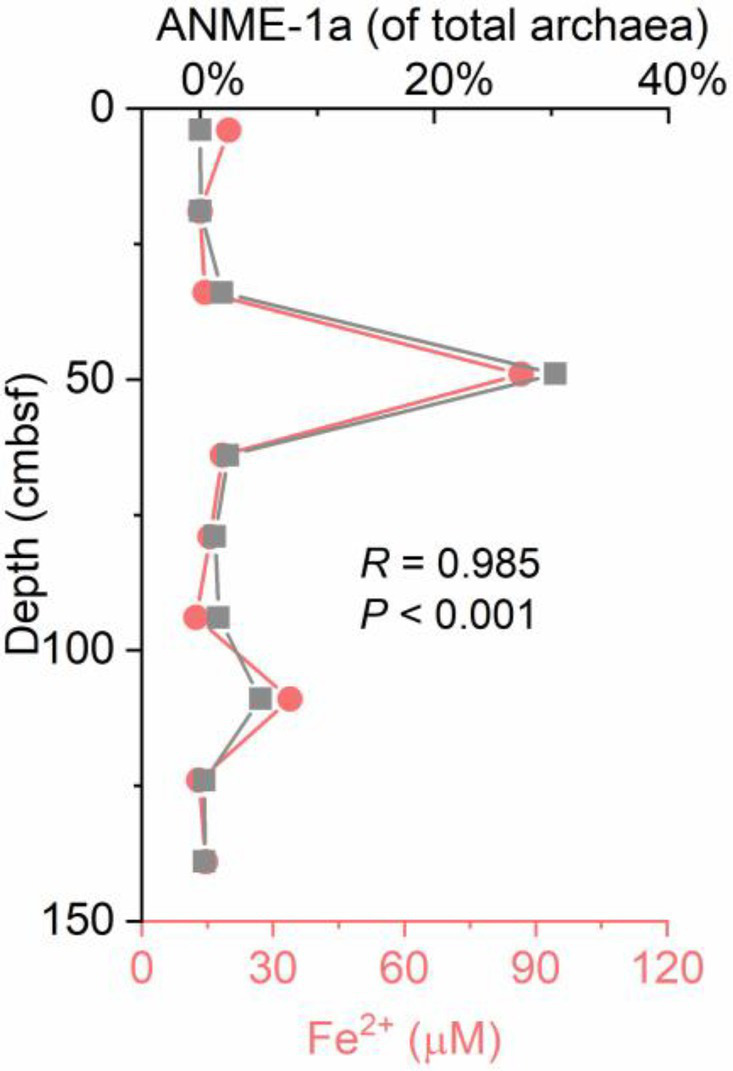
Profiles showing the relative abundance of ANME-1a and corresponding concentrations of pore water dissolved iron from gravity core GC2020-02.

Geochemical characteristics below the SMTZ, such as depleted sulfate, high contents of buried reactive iron oxides, and the presence of methane, are the preceding conditions for Fe-AOM (Riedinger et al., 2014). Multiple lines of evidence in mineralogy and geochemistry have suggested that iron-coupled AOM is present in the OT (Sun et al., 2015, 2019; Peng et al., 2017). A sufficient Fe supply from the adjacent hydrothermal systems of the OT provided an ideal environment for metal-reducing microorganisms capable of using Fe oxides to oxidize methane (Sun et al., 2019). In the estimated SMTZ sediments, a similar trend between the *mcrA* gene numbers and the concentration of dissolved iron (Fe^2+^) was detected in the samples. Specifically, the *mcrA* gene numbers showed their highest abundance in the peak of dissolved iron (Fe^2+^) at 49 cm (Supplementary Figure S4). Another peak of Fe^2+^ concentration was found at 109 cm, with elevated *mcrA* gene numbers (Supplementary Figure S4). These results implied that methane-metabolizing archaea could be involved in iron reduction in the OT cold seep. In marine sediments, AOM is always reported to occur in deep methanogenic zones with depleted sulfate *via* iron reduction (Liang et al., 2019). However, it should be noted that several studies have suggested that iron oxides could stimulate AOM even in the sulfate-containing zone (Beal et al., 2009; Sivan et al., 2014). It was believed that iron oxides could indirectly stimulate sulfate-driven AOM *via* a “cryptic” sulfur cycle, as suggested by Holmkvist et al. (2011). Holmkvist et al. (2011) noted that the oxidation of hydrogen sulfide to sulfide oxidation intermediates is coupled with the reduction in iron oxides. Then, these intermediates are disproportionated to sulfate and sulfide in what is termed a “cryptic” sulfur cycle. In the OT seep sediments, the possible mechanism of Fe-AOM remains unclear. More geochemical evidence and microbial incubation experiments should be conducted to provide direct evidence for Fe-AOM and determine its possible mechanism. It was interesting to find that the depthwise distribution of the relative abundance of ANME-1a showed a strong positive covariance with the dissolved Fe(II) pore water concentrations (*R* = 0.99; *p* < 0.001; Figure 8), suggesting that ANME-1a might play an important role in methane oxidation coupled with Fe(III) reduction. This speculation was further confirmed by RDA of ANME-1a, which was strongly correlated with Fe^2+^ (Figure 7).

### Co-occurrence Analysis

Network co-occurrence analysis was used to explore ecologically relevant relationships between methanogens/ANMEs and other microbial taxa. According to the analysis, most ASVs belonging to ANME clades, including ANME-1a, ANME-1b, ANME-2c, and methanogen groups (Methanofastidiosales and Methanomassiliicoccales), were associated with JS1 and Bathy-1. JS1 populations were particularly distributed in many methane-containing environments, such as cold seep sediments, methane hydrate-bearing sediments, and hydrothermal vents (Inagaki et al., 2002; Teske et al., 2002; Pachiadaki et al., 2011; Lee et al., 2013), and were proposed as a possible partner in AOM. In addition, members of the JS1 group are likely to be heterotrophic bacteria (Nobu et al., 2016) and can catabolize acetate and glucose as substrates (Webster et al., 2006, 2011). Metagenomic analysis showed that some members of the JS1 group might be involved in the degradation of hydrocarbon compounds (Liu et al., 2019). Microbes in Bathyarchaeota are heterotrophs capable of degrading various organic matter (e.g., acetate, detrital proteins, and plant-derived carbohydrates) as growth substrates for biosynthesis and energy production (Webster et al., 2010; Meng et al., 2014; Lazar et al., 2016). Moreover, Bathyarchaeota have been shown to contain genes involved in methane metabolism (Evans et al., 2015) and acetogenesis. Previous studies revealed a positive correlation between bathyarchaeotal abundance and TOC content, suggesting that Bathyarchaeota play important roles in organic carbon remineralization (Yu et al., 2017). The ecological interactions among microbes in the OT cold seep sediments are consistent with the fact that the methanogen/ANME population and some heterotrophic microbial groups could interact metabolically through an anaerobic food chain.

## Conclusion

In the present study, we investigated methane-metabolizing microbial communities in a sediment core from an active cold seep of the Okinawa Trough. Molecular results indicated that ANME-1 (ANME-1a and ANME-1b), ANME-2 (ANME-2a/b and ANME-2c), and ANME-3 co-occurred at the cold seep site investigated. Niche separation of ANME subgroups was also observed in the seep sediments of the OT. ANME-3 was dominant in the upper sediments under sulfide-rich conditions, while ANME-1 tended to dominate anoxic, sulfate-depleted, and highly sulfidic sediments. In addition, H_2_-dependent methylotrophic Methanofastidiosales and Methanomassiliicoccales were highly abundant in the estimated SMTZ in the OT cold seep sediments. RDA analyses indicated that dissolved ions and sulfate were the significant factors controlling the composition of the methane-metabolizing community. Moreover, the relative abundance of ANME-1a was strongly correlated with the profile of pore water Fe^2+^, suggesting that ANME-1a is the most promising ANME phylotype for participating in the potential Fe-AOM.

## Data Availability Statement

The 16S rRNA gene sequencing data presented in the study are deposited in the Sequence Read Archive (SRA) database, accession number PRJNA781813. The sequences of the mcrA gene clone library are submitted to GenBank, with assigned accession numbers ON045010-ON045071. The names of the repository/repositories and accession number(s) can be found in the article/**Supplementary Material**.

## Author Contributions

YC, NW, and ZS conceived the study and designed the experiments. CX determined the physicochemical parameters. YC performed the experiments and analyzed the data. YC, NW, and ZS wrote the manuscript. YC, CX, YX, JL, SQ, and YZho processed the core sediments. CL, YZhe, XZ, WG, HC, and BZ edited and approved the final manuscript. All authors contributed to the article and approved the submitted version.

## Funding

This work was supported by the NSFC Major Research Plan on West-Pacific Earth System Multispheric Interactions (project number: 91858208), the National Natural Science Foundation of China (project numbers: 42106137, 42176057, and 41906068), and the Marine Geological Survey Program of China Geological Survey (project number: DD20221707).

## Conflict of Interest

The authors declare that the research was conducted in the absence of any commercial or financial relationships that could be construed as a potential conflict of interest.

## Publisher’s Note

All claims expressed in this article are solely those of the authors and do not necessarily represent those of their affiliated organizations, or those of the publisher, the editors and the reviewers. Any product that may be evaluated in this article, or claim that may be made by its manufacturer, is not guaranteed or endorsed by the publisher.

## Footnotes

^1^ https://www.ncbi.nlm.nih.gov/tools/primer-blast/
