# Supplementary material for: Diversity of Anaerobic Methane Oxidizers in the Cold Seep Sediments of the Okinawa Trough
Source: Front Microbiol. 2022 Apr 14;13:819187. doi: 10.3389/fmicb.2022.819187 (PMC9048799; doi:10.3389/fmicb.2022.819187)
Supplement: Supplementary file 1 [file Data_Sheet_1.docx]

Fig.S1 Relative abundance of major archaeal (A) and bacterial (B) community (with relative abundance of >1% in at least one sample) in the sediments of site GC2020-02





Fig. S2 The composition of putative ANME/methanogens obtained from the 16S rRNA gene sequences of the V3–V4 region in the GC2020-02 core.


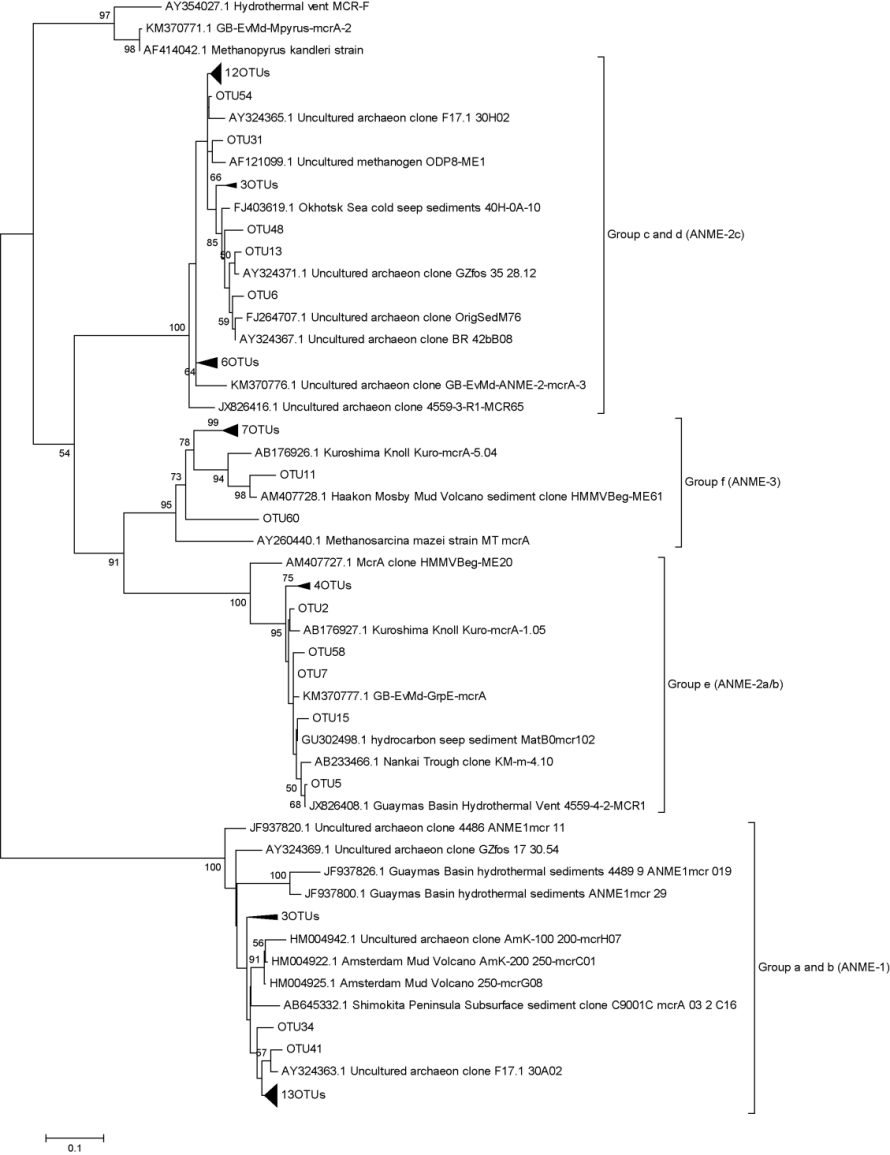


Fig. S3 Maximum likelihood phylogenetic tree of *mcrA* sequences from the respective sequences of OTUs. Bootstrap values greater than 50% for 1000 resamplings are shown close to the nodes. *Methanopyrus kanderi stain* served as outgroup references.


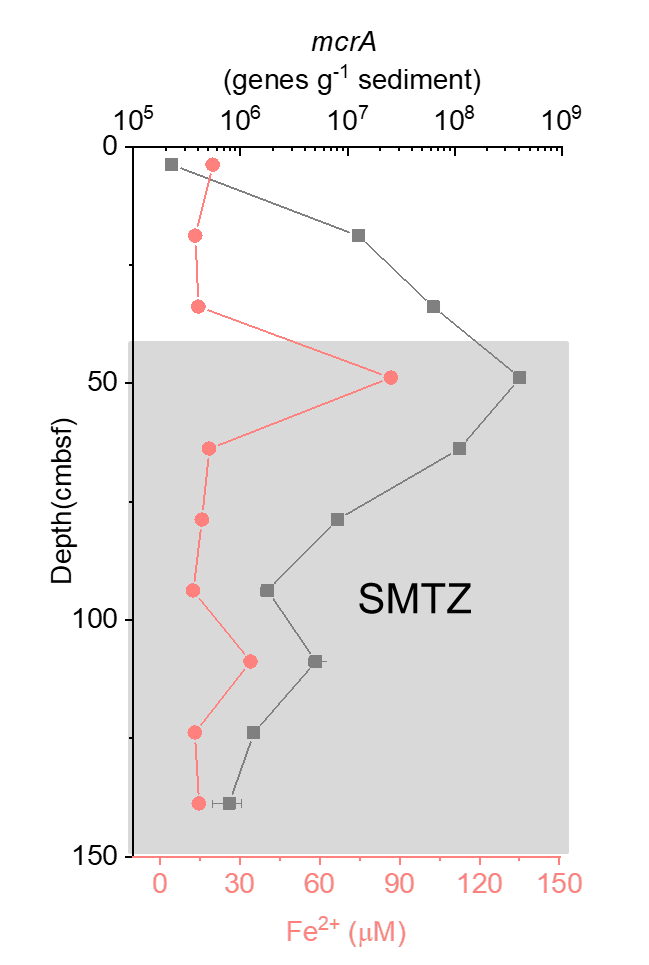


Fig. S4 Profiles showing the distribution of the *mcrA* gene numbers and corresponding concentrations of pore water dissolved iron from gravity core GC2020-02

Table S1 PCR primers assay conditions and efficiencies used for qPCR of 16S rRNA genes and the key functional gene *dsr*A.

| Name | Target group | Sequence(5`-3`) | Annealing  temperature | Average  efficiency | R^2^ | References |
| --- | --- | --- | --- | --- | --- | --- |
| 338F | 16S rRNA gene of Bacteria | ACTCCTACGGGAGGCAGCA | 58◦C | 99.07% | 0.998 | (Peiffer *et al.*, 2013) |
| 806R |  | GGACTACHVGGGTWTCTAAT |  |  |  |  |
| Arch519 | 16S rRNA gene of Archaea | CAGCCGCCGCGGTAA | 58◦C | 90.79% | 0.997 | (Coolen *et al.*, 2004) |
| Arch806 |  | GTGCTCCCCCGCCAATTCCT |  |  |  |  |
| mlasF | methanogen/ANME | GGTGGTGTMGGDTTCACMCARTA | 55◦C | 85.16% | 0.982 | (Steinberg et al., 2008; Mori et al., 2012) |
| ME2mod |  | TCATBGCRTAGTTNGGRTAGT |  |  |  |  |
| mcrA_ab_fw | *mcrA* gene of ANME-1 | AYGACCAGTTGTGGTTCGGAACGT | 62◦C | 100.85% | 0.994 | (Miyazaki et al., 2009) |
| mcrA-ab-rv |  | TCCATGTTSARCTTGTCGCCCTTT |  |  |  |  |
| mcrA-f-fw | *mcrA* gene of ANME-3 | AAGGAYATYRSAACCGAATC | 52◦C | 85.79% | 0.996 | (Miyazaki et al., 2009) |
| mcrA-f-rv |  | TTGAAAGGTACCATSSKGAAAGACC |  |  |  |  |
| DSRp2060F | *dsrB* | CAA CAT CGT YCA YAC CCA GGG | 55◦C | 98.80% | 0.994 | (Geets et al., 2006) |
| DSR-4R |  | GTG TAG CAG TTA CCG CA |  |  |  |  |

Table S2 The richness estimator, diversity index and Goods_coverage of archaeal and bacterial communities

| Depth (cm) |  |  | Archaea |  |  |  |  |  | Bacteria |  |  |
| --- | --- | --- | --- | --- | --- | --- | --- | --- | --- | --- | --- |
|  | Reads | ASVs | Chao1 | Shannon | Goods_coverage |  | Reads | ASVs | Chao1 | Shannon | Goods_coverage |
| 3.75 | 37654 | 3521 | 3536.47 | 10.46 | 99.60% |  | 72530 | 4370 | 5614.9 | 10.8901 | 94.27% |
| 18.75 | 42854 | 3027 | 3101.90 | 9.76 | 99.12% |  | 26512 | 3653 | 3666.38 | 10.5424 | 99.19% |
| 33.75 | 40983 | 2457 | 2514.13 | 9.02 | 99.24% |  | 84337 | 3342 | 4167.81 | 10.0807 | 95.92% |
| 48.75 | 56890 | 1021 | 1092.38 | 6.52 | 99.58% |  | 46970 | 1659 | 1942.84 | 7.28566 | 98.16% |
| 63.75 | 47712 | 933 | 997.49 | 6.38 | 99.64% |  | 95746 | 1628 | 2035.16 | 7.42981 | 98.08% |
| 78.75 | 56464 | 2529 | 2822.76 | 8.26 | 98.39% |  | 59470 | 2390 | 2948.08 | 8.57034 | 97.02% |
| 93.75 | 49674 | 1510 | 1641.86 | 7.24 | 99.26% |  | 106462 | 2806 | 3458.68 | 9.0317 | 96.60% |
| 108.75 | 52508 | 2583 | 2852.54 | 8.61 | 98.46% |  | 62694 | 3162 | 3985 | 9.62757 | 95.84% |
| 123.75 | 49023 | 1604 | 1772.20 | 7.77 | 99.21% |  | 110476 | 2731 | 3398.19 | 9.06348 | 96.66% |
| 138.75 | 55024 | 3554 | 3924.11 | 9.28 | 97.68% |  | 84248 | 3271 | 4330.07 | 9.35946 | 95.34% |

Table S3 Dominant methane-oxidizing archaea groups in the cold seep of Okinawa Trough and other cold seeps

| Habitat | Site Description | ANME groups | References |
| --- | --- | --- | --- |
| the Hydrate Ridge and Black Sea | *Beggiatoa* mats, discrete methane hydrate layers occur and strong degassing from decomposing hydrates was observed | ANME-1a, ANME-1b, ANME-2a, ANME-2b, ANME-2c, ANME-3 | (Knittel *et al.*, 2005) |
| gas hydrate-bearing  sediments in the South China Sea | contained some lightyellow authigenic carbonates and gas hydrate | ANME-1a, ANME-1b, ANME-2a, ANME-2c | (Cui *et al.*, 2019) |
| Sonora Margin cold seeps | white *Beggiatoa* mats, gas bubbles were noted | ANME-1, ANME-2a/b, ANME-2c, ANME-3 | (Vigneron *et al.*, 2013) |
| the Haakon Mosby Mud Volcano | dense *Beggiatoa* mats, mud expulsion, sulfidic sediments, dense accumulations of thread-like  tubeworms (Siboglinidae) | ANME-2a, ANME-3 | (Lösekann *et al.*, 2007) |
| methane seepage off the coast of the Tuscan Island Elba | methane emission spots, white mats of sulfur-oxidizing bacteria | ANME-1a/b, ANME-2a/b/c, ANME-3 | (Ruff *et al.*, 2016) |
| Haima cold seep area | numerous gas bubbles, a strong smell of hydrogen sulfide | ANME-1b, ANME-2ab, ANME-2c, ANME-2d | (Niu *et al.*, 2017) |
| Scotian Basin cold seep | gas hydrates, gas bubbles, strong sulfifide odor processing | ANME-1, ANME-2ab, ANME-2c, ANME-3 | (Dong *et al.*, 2020) |
| cold seep sediments of Nyegga pockmarks | methane hydrates, upward-migrating methane fluids, a white microbial mat | ANME-1, ANME-2ab, ANME-2c | (Roalkvam *et al.*, 2011) |
| cold seeps of Northern South China Sea | large amounts of methane gas hydrates, authigenic carbonates and fossil shells | ANME-1, ANME-2a/b, ANME-2c, ANME-2d, ANME-3 | (Zhang *et al.*, 2020) |
| Okinawa Trough cold seep | gas bubble emission, a strong sulfide odor, carbonate gravels and giant clam shells | ANME-1, ANME-2a/b, ANME-2c, ANME-3 | This study |

**Reference:**

Coolen, M.J., Hopmans, E.C., Rijpstra, W.I.C., Muyzer, G., Schouten, S., Volkman, J.K., et al. (2004). Evolution of the methane cycle in Ace Lake (Antarctica) during the Holocene: response of methanogens and methanotrophs to environmental change. *Org. Geochem.* 35(10)**,** 1151-1167. doi: 10.1016/j.orggeochem.2004.06.009

Cui, H., Su, X., Chen, F., Holland, M., Yang, S., Liang, J., et al. (2019). Microbial diversity of two cold seep systems in gas hydrate-bearing sediments in the South China Sea. *Mar. Environ. Res.* 144:230-239. doi: 10.1016/j.marenvres.2019.01.009.

Dong, X., Rattray, J., Campbell, D., Webb, J., Chakraborty, A., Adebayo, O., et al. (2020). Thermogenic hydrocarbon biodegradation by diverse depth-stratified microbial populations at a Scotian Basin cold seep. *Nat. Commun.* 11**,** 5825. doi: 10.1038/s41467-020-19648-2.

Geets, J., Borremans, B., Diels, L., Springael, D., Vangronsveld, J., van der Lelie, D., et al. (2006). DsrB gene-based DGGE for community and diversity surveys of sulfate-reducing bacteria. *J. Microbiol. Methods*, 66(2)**,** 194-205. doi: 10.1016/j.mimet.2005.11.002.

Knittel, K., Loesekann, T., Boetius, A., Kort, R., and Amann, R. (2005). Diversity and Distribution of Methanotrophic Archaea at Cold Seeps. *Appl. Environ. Microbiol.* 71**,** 467-479. doi: 10.1128/AEM.71.1.467-479.2005.

Lösekann, T., Knittel, K., Nadalig, T., Fuchs, B., Niemann, H., Boetius, A., et al. (2007). Diversity and abundance of aerobic and anaerobic methane oxidizers at the Haakon Mosby Mud Volcano, Barents Sea. *Appl. Environ. Microbiol.* 73(10)**,** 3348-3362. doi: 10.1128/aem.00016-07.

Miyazaki, J., Higa, R., Toki, T., Ashi, J., Tsunogai, U., Nunoura, T., et al. (2009). Molecular characterization of potential nitrogen fixation by anaerobic methane-oxidizing archaea in the methane seep sediments at the number 8 Kumano Knoll in the Kumano Basin, offshore of Japan. *Appl. Environ. Microbiol.* 75(22)**,** 7153-7162.

Mori, K., Iino, T., Suzuki, K.-I., Yamaguchi, K., and Kamagata, Y. (2012). Aceticlastic and NaCl-Requiring Methanogen "Methanosaeta pelagica" sp nov., Isolated from Marine Tidal Flat Sediment. *Appl. Environ. Microbiol.* 78**,** 3416-3423. doi: 10.1128/AEM.07484-11.

Niu, M., Fan, X., Zhuang, G., Liang, Q., and Wang, F.J.F.m.e. (2017). Methane-metabolizing microbial communities in sediments of the Haima cold seep area, northwest slope of the South China Sea. *FEMS Microbiol. Ecol.* 93(9). doi: 10.1093/femsec/fix101.

Peiffer, J.A., Spor, A., Koren, O., Jin, Z., Tringe, S.G., Dangl, J.L., et al. (2013). Diversity and heritability of the maize rhizosphere microbiome under field conditions. *Proc. Natl. Acad. Sci. U.S.A.* 110(16)**,** 6548-6553. doi: 10.1073/pnas.1302837110.

Roalkvam, I., Jørgensen, S.L., Chen, Y., Stokke, R., Dahle, H., Hocking, W.P., et al. (2011). New insight into stratification of anaerobic methanotrophs in cold seep sediments. *FEMS Microbiol. Ecol.* 78(2)**,** 233-243. doi: 10.1111/j.1574-6941.2011.01153.x.

Ruff, S.E., Kuhfuss, H., Wegener, G., Lott, C., Ramette, A., Wiedling, J., et al. (2016). Methane seep in shallow-water permeable sediment harbors high diversity of anaerobic methanotrophic communities, Elba, Italy. *Front. Microbiol.* 7**,** 374. doi: 10.3389/fmicb.2016.00374.

Steinberg, L.M., Regan, J.M.J.A., and microbiology, e. (2008). Phylogenetic comparison of the methanogenic communities from an acidic, oligotrophic fen and an anaerobic digester treating municipal wastewater sludge. *Appl. Environ. Microbiol.* 74(21), 6663-6671. doi: 10.1128/AEM.00553-08.

Vigneron, A., Cruaud, P., Pignet, P., Caprais, J.C., Cambon-Bonavita, M.A., Godfroy, A., et al. (2013). Archaeal and anaerobic methane oxidizer communities in the Sonora Margin cold seeps, Guaymas Basin (Gulf of California). *ISME J.* 7(8)**,** 1595-1608. doi: 10.1038/ismej.2013.18.

Zhang, T., Xiao, X., Chen, S., Zhao, J., Chen, Z., Feng, J., et al. (2020). Active Anaerobic Archaeal Methanotrophs in Recently Emerged Cold Seeps of Northern South China Sea. *Front. Microbiol.* 11:612135. doi: 10.3389/fmicb.2020.612135. eCollection 2020.
